# Post-stroke Intranasal (+)-Naloxone Delivery Reduces Microglial Activation and Improves Behavioral Recovery from Ischemic Injury

**DOI:** 10.1523/ENEURO.0395-17.2018

**Published:** 2018-04-18

**Authors:** Jenni E. Anttila, Katrina Albert, Emily S. Wires, Kert Mätlik, Lisa C. Loram, Linda R. Watkins, Kenner C. Rice, Yun Wang, Brandon K. Harvey, Mikko Airavaara

**Affiliations:** 1 Institute of Biotechnology, HiLIFE Unit, University of Helsinki, 00014, Finland; 2 Intramural Research Program, National Institute on Drug Abuse, IRP, NIH, Baltimore, MD 21224; 3 Medicum, Department of Pharmacology, University of Helsinki, 00014, Finland; 4 Department of Psychology & Neuroscience, University of Colorado, Boulder, CO 80309; 5 Center for Neuropsychiatric Research, National Health Research Institutes, Zhunan, 35053, Taiwan

**Keywords:** Microglia, middle cerebral artery occlusion, naloxone, NF-κB, secondary injury, stroke

## Abstract

Ischemic stroke is the leading cause of disability, and effective therapeutic strategies are needed to promote complete recovery. Neuroinflammation plays a significant role in stroke pathophysiology, and there is limited understanding of how it affects recovery. The aim of this study was to characterize the spatiotemporal expression profile of microglial activation and whether dampening microglial/macrophage activation post-stroke facilitates the recovery. For dampening microglial/macrophage activation, we chose intranasal administration of naloxone, a drug that is already in clinical use for opioid overdose and is known to decrease microglia/macrophage activation. We characterized the temporal progression of microglia/macrophage activation following cortical ischemic injury in rat and found the peak activation in cortex 7 d post-stroke. Unexpectedly, there was a chronic expression of phagocytic cells in the thalamus associated with neuronal loss. (+)-Naloxone, an enantiomer that reduces microglial activation without antagonizing opioid receptors, was administered intranasally starting 1 d post-stroke and continuing for 7 d. (+)-Naloxone treatment decreased microglia/macrophage activation in the striatum and thalamus, promoted behavioral recovery during the 14-d monitoring period, and reduced neuronal death in the lesioned cortex and ipsilateral thalamus. Our results are the first to show that post-stroke intranasal (+)-naloxone administration promotes short-term functional recovery and reduces microglia/macrophage activation. Therefore, (+)-naloxone is a promising drug for the treatment of ischemic stroke, and further studies should be conducted.

## Significance Statement

Ischemic stroke is one of the leading causes of adult disability, and new drug treatments are needed, as there is no drug that promotes recovery. Neuroinflammation is suggested to play a role in the recovery process. Naloxone is a drug used clinically to treat opioid overdose. Its opioid receptor inactive form, (+)-naloxone, is known to reduce the activation of microglia, the immune cells of the brain. We show for the first time that repeated dosing of intranasal (+)-naloxone starting 1 d after stroke promotes short-term recovery in rats and reduces microglial activation and neuronal loss in the stroke brain. Our finding could be important for future stroke treatment and encourages further testing of (+)-naloxone in stroke patients.

## Introduction

Approximately 10 million patients survive a stroke each year; however, there are currently no pharmacological options to promote recovery. Neurologic deficits including impaired use of contralateral limbs, sensory and cognitive deficits, and problems in speaking are the primary cause of disability and remain without effective treatment. Despite increased knowledge of the cellular and molecular mechanisms that mediate damage and recovery after stroke, the development of new drugs for stroke has been unsuccessful (Dirnagl, 2012). Most studies have focused on neuroprotection, and therefore, there is a great need to find novel drug targets and develop new treatments that would improve the recovery from ischemic brain injury by targeting postischemic pathologic mechanisms such as inflammation. Modulation of brain inflammatory cascades following stroke is recognized as a viable therapeutic strategy to promote functional recovery from ischemic brain injury (Endres et al., 2008). However, very few studies have taken this approach, and little is known about recovery from stroke in relation to neuroinflammation.

**Figure 1.**
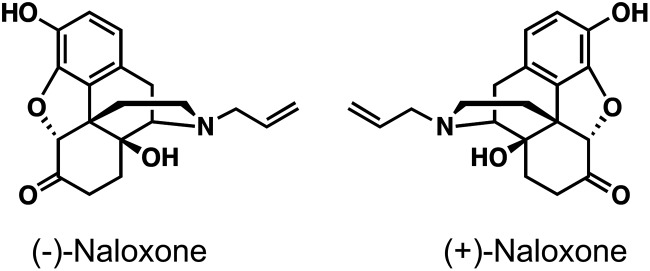
**Chemical structure of (–)-naloxone and (+)-naloxone enantiomers.** *K_i_* of (–)-naloxone for antagonizing opioid receptors is in the 1 nM range, whereas (+)-naloxone has a very low affinity for opioid receptors, with a *K_i_* of 10,000 nM (Iijima et al., 1978).

The drug naloxone, an opiate antagonist, has been clinically used for opioid overdose for nearly 50 years, has anti-inflammatory properties, and can attenuate microglial activation. Naloxone has two stereoisomers, the (–) and (+) enantiomers (Fig. 1). (–)-Naloxone has a high affinity for antagonizing µ, δ, and κ opioid receptors, whereas (+)-naloxone has a very low affinity for opioid receptors (Iijima et al., 1978). Clinical reports indicate that intravenously administered (–)-naloxone ameliorates neurologic deficits in acute stroke (Baskin and Hosobuchi, 1981; Jabaily and Davis, 1984). Studies examining the neuroprotective effect of (–)-naloxone in rat focal cerebral ischemia also showed a significant reduction in neuronal loss and inflammation (Chen et al., 2001; Liao et al., 2003), but (+)-naloxone did not affect infarction volume when administered before ischemia. These differences between (–) and (+) isoforms suggest a role for opioid receptor antagonism in the neuroprotective effect (Liao et al., 2003). However, both enantiomers have been shown to reduce the number of activated microglia in a rat model of neuropathic pain (Hutchinson et al., 2008) and to inhibit superoxide production from microglia, and subsequent neurodegeneration, by interacting with NADPH oxidase (Liu et al., 2002; Qin et al., 2005; Wang et al., 2012). Also, both naloxone enantiomers reduce lipopolysaccharide (LPS)-induced microglial activation and protect neurons from LPS-induced neurodegeneration (Liu et al., 2000a, b, c). LPS signaling in microglia/macrophages requires Toll-like receptor 4 (TLR4) that is expressed abundantly on microglia (Lehnardt et al., 2003). Both (–)- and (+)-naloxone enantiomers decrease LPS-induced TLR4 signaling (Hutchinson et al., 2008; Wang et al., 2016) and both have been shown to antagonize the TLR4 coreceptor MD2 (Hutchinson et al., 2012). TLR4 activation leads to activation of NF-κB and production of pro-inflammatory cytokines, interferons, and reactive oxygen/nitrogen species. TLR4 deficiency is neuroprotective in ischemic stroke in mice (Kilic et al., 2008), and increased TLR4 expression is associated with more severe stroke in patients (Yang et al., 2008).

Collectively, these studies suggest that naloxone can modulate immune and glial responses and has therapeutic potential in stroke. However, its effects on post-stroke microglial activation and recovery have not been studied. Because (+) and (–) forms have similar efficacy in reducing microglial activation, the advantage of the (+)-form is that side effects from antagonizing opioid receptors can be avoided. Thus, we aimed to study whether prolonged post-stroke administration of (+)-naloxone in rats would promote recovery and whether this recovery is associated with altered levels of activated microglia/macrophages. Furthermore, we took an approach of intranasal administration, since recent advances with intranasal naloxone formulations have provided excellent bioavailability and enabled easy administration in patients. We first characterized the neuroinflammatory response following focal cortical ischemia-reperfusion injury, since it is not well studied. As microglia/macrophages showed activation already on day 2 post-stroke in the ischemic cortex and maximum activation was observed at day 7, we administered (+)-naloxone during this accumulation period. Here we show how post-stroke intranasal (+)-naloxone promoted behavioral recovery during the short 14-d testing period and decreased microglia/macrophage activation and neuronal loss.

## Materials and Methods

### Animals

Adult male Sprague-Dawley rats (200–250 g; Charles River) were maintained under a 12-h light-dark cycle. Food and water were freely available in the home cage. Experimental procedures were approved by the NIDA Animal Care and Use Committee or by the National Animal Experiment Board of Finland (protocol approval number ESAVI/5459/04.10.03/2011) and followed the guidelines of the “Guide for the Care and Use of Laboratory Animals” (National Institutes of Health publication, 1996) and local laws and regulations.

### Cortical stroke model in rats with distal middle cerebral artery occlusion

To model focal cortical ischemic stroke in rats, the three-vessel occlusion method was used. In this model, the stroke damage is restricted to the cortex, and the relative size of the stroke is close to that of an average human stroke (Delavaran et al., 2013). Ligation of the right middle cerebral artery (MCA) and common carotid arteries (CCAs) bilaterally was performed as described previously (Chen et al., 1986; Airavaara et al., 2009, 2010; Harvey et al., 2011). Briefly, rats were anesthetized with chloral hydrate 0.4 g/kg intraperitoneally (i.p.). The bilateral CCAs were identified and isolated through a ventral midline cervical incision. The rats were placed in a stereotaxic apparatus, and a craniotomy was performed to expose the right MCA. The MCA was ligated with a 10-0 suture, and bilateral CCAs were ligated with nontraumatic arterial clamps. After 60 or 90 min of ischemia, the suture around the MCA and arterial clips on CCAs were removed to begin reperfusion. After recovery from anesthesia, rats were returned to their home cage. Body temperature during and after surgery was maintained at 37°C.

### Intranasal administration of (–)-naloxone and (+)-naloxone

(–)-Naloxone and (+)-naloxone were synthesized in the laboratory of Dr. Kenner Rice (NIDA IRP, NIH). Dosing was estimated from a previously published study (Hutchinson et al., 2008). Drugs were administered intranasally under isoflurane anesthesia (Anesthesia Auto Flow System; E-Z Anesthesia) starting from post-stroke day 1 and were continued at 12-h intervals for 7 d, a total of 14 times. Briefly, animals were placed in the induction chamber for 1–1.5 min, and 5% isoflurane was delivered at 1000 cc/min. Animals were transferred to the nose cone where they received 1.5% isoflurane delivered at 500 cc/min for 30 s before intranasal naloxone delivery. To ensure maximal delivery, animals remained in supine position during naloxone administration. When the animals regained consciousness, they were returned to the home cage. Naloxone solution was prepared fresh daily in sterile ultrapure water and stored at room temperature between administrations. Using a Rainin LTS L-20 pipette and sterile pipette tips, 10 µl drug or vehicle was administered into each nostril as described (Luo et al., 2013).

### Implantation of mini-osmotic pumps for continuous (+)-naloxone delivery

Two days after distal middle cerebral artery occlusion (dMCAo) surgery, the rats were anaesthetized with chloral hydrate (0.4 mg/kg i.p.) for mini-osmotic pump implantation (Alzet; model 2002). The pump was implanted under the skin, and the catheter was inserted into the right ventricle. The pumps were filled with vehicle (sterile, ultrapure water) or (+)-naloxone 96 mg/ml. The pumping rate was 0.5 µl/h, and the pumps were left in place for 12 d.

### Behavioral analysis

The elevated body swing test for body asymmetry (Borlongan et al., 1998), modified Bederson’s score (Bederson et al., 1986), and measurement of locomotor activity were performed as previously described (Airavaara et al., 2009, 2010). Briefly, body asymmetry was analyzed from 20 consecutive trials by suspending the rats 20 cm above the testing table by lifting their tails and counting the frequency of initial turnings of the head or upper body contralateral to the ischemic side (the maximum impairment in stroke animals is 20 contralateral turns, whereas naive animals turn in each direction with equal frequency). For Bederson’s score, neurologic deficits were scored using the following criteria: 0, rats extend both forelimbs straight when lifted by the tail, no observable deficit; 1, rat keeps the one forelimb to the breast and extends the other forelimb straight when lifted by the tail; 2, rat shows decreased resistance to lateral push in addition to behavior in score 1; 3, rat twists the upper half of the body when lifted by the tail in addition to behavior in other scores. Locomotor activity was measured for 24 h by placing the rat in a 42 × 42 × 31-cm Plexiglas box with an infrared activity monitor (Accuscan). All behavioral assessments were performed in a blinded manner, and the experimenter did not know group allocation. Body asymmetry and Bederson’s score were assessed at the middle of the day, so that the animals had fully recovered from isoflurane anesthesia needed for the period of intranasal administration in the morning and evening.

### Histology

The rats were deeply anaesthetized with pentobarbital (90 mg/kg i.p.) and transcardially perfused with 200 ml saline followed by 500 ml of 4% paraformaldehyde. Brains were processed for either paraffin or free-floating sections and stained with anti-Iba1, anti-CD68, anti-NeuN, anti-GFAP, or anti-MBP antibodies.

### Immunostaining of paraffin sections

Brains were postfixed in 4% paraformaldehyde for 2 d, dehydrated in a series of ethanol and xylene, and embedded in paraffin. Brains were cut into 5-µm-thick sagittal sections using a Leica HM355S microtome and mounted on Superfrost Plus slides (Thermo Fisher Scientific). Sections were deparaffinized, and antigen retrieval was performed by heating in 0.05% citraconic anhydride (Sigma-Aldrich), pH 7.4. Endogenous peroxidase activity was blocked with 0.6% hydrogen peroxide (Sigma-Aldrich), and nonspecific antibody binding was blocked with 1.5% normal goat or horse serum (Vector Laboratories), followed by incubation with primary antibody (rabbit anti-Iba1 1:1000, #019-19741, RRID:AB_839504, Wako; mouse anti-CD68 1:500, #MCA341R, RRID:AB_2291300, AbD Serotec; mouse anti-GFAP 1:1000, #MAB360, RRID:AB_2109815, Millipore; mouse anti-NeuN 1:200, #MAB377, RRID:AB_2298772, Millipore; and rabbit anti-MBP 1:500, #ab40390, RRID:AB_1141521, Abcam) at 4°C overnight. The next day, sections were incubated with secondary antibody (goat anti-rabbit, RRID:AB_2336820, or horse anti-mouse, RRID:AB_2336811, biotinylated secondary antibody 1:200, Vector Laboratories) followed by incubation with avidin-biotin complex (ABC kit, Vector Laboratories). Color was developed using peroxidase reaction with 3′,3′-diaminobenzidine (DAB; RRID:AB_2336382, Vector Laboratories). Cresyl violet staining was used as background staining with anti-CD68. For immunofluorescence staining, goat anti-rabbit Alexa Fluor 488 (1:500, #A11034, RRID:AB_2576217, Life Technologies) and goat anti-mouse Alexa Fluor 568 (1:500, #A11004, RRID:AB_2534072, Life Technologies) were used as secondary antibodies.

### Immunostaining of free-floating sections

Brains were dehydrated in 30% sucrose at 4°C and sectioned coronally into 40-μm-thick slices using a Leica CM3050 Cryostat. Sections were taken from 2.1 to –1.0 mm (striatum) and –2.4 to –4.0 mm (thalamus) relative to bregma, then stored in 1× PBS for short-term storage or cryopreservant for long-term storage (20% glycerol, 2% DMSO in 1× PBS). Sections were blocked with 0.3% hydrogen peroxidase and 4% BSA (Sigma-Aldrich) + 0.3% Triton X-100 (Sigma-Aldrich), then incubated with primary antibody (rabbit anti-Iba1 1:2000, RRID:AB_839504; mouse anti-NeuN 1:1000, RRID:AB_2298772) overnight at 4°C. The next day, sections were incubated with secondary antibody, followed by incubation with avidin-biotin complex and DAB as above.

### Unbiased stereological counting of Iba1^+^ cells in the striatum

Iba1-positive (Iba1^+^) cells in the striatum were counted from 40-µm-thick free-floating sections using unbiased stereology with a stereomicroscope (Olympus BX51) and StereoInvestigator 6 program (MBF Bioscience) as previously described (Mijatovic et al., 2007). The optical fractionator method, which involves a three-dimensional probe for counting the cell of interest by randomly and systematically sampling each section, was used. It is unbiased, since it does not involve cell size and shape, and is unaffected by tissue processing. Cell population is computed from the volume fraction. The volume fraction consists of information on the thickness of tissue sampled, the number of sections sampled, and the area in each section sampled. Three 40-µm-thick coronal slices were selected based on their relative location to bregma (0.2, –0.26, and –0.4/–0.5 mm) to obtain results relatively free of bias in the distribution of the cells. Only Iba1^+^ cells with clear microglia morphology were counted. The contralateral and ipsilateral striata were first traced, and then the area was analyzed for each slice. Approximately 60–80 randomly selected sites per area/slice were analyzed to ensure accuracy and minimize error.

### Analysis of CD68^+^, Iba1^+^, and NeuN^+^ cells in the thalamus

The number of CD68^+^, Iba1^+^, and NeuN^+^ cells in the thalamus was analyzed using Image-Pro Analyzer 7.0 program. Slides were scanned with a 3DHISTECH Pannoramic 250 FLASH II digital slide scanner (scanning service provided by the Institute of Biotechnology, University of Helsinki; http://www.biocenter.helsinki.fi/bi/histoscanner/index.html), and 10× magnification images of the thalamus were taken with Pannoramic Viewer version 1.15.3. To estimate the number of immunopositive cells in the ipsilateral thalamus, the thalamus was traced and the Iba1 immunoreactive area was counted from 3–6 coronal sections (in 3 rats, there were only 2 sections counted). The object count of NeuN^+^ cells in the thalamus was counted from 4–6 coronal sections per brain. Sections were taken every 300–400 µm between –2.4 and –4.0 mm relative to bregma, and an average for each brain was used for further analysis. The average number of NeuN^+^ cells and the Iba1 immunoreactive area are expressed as a percentage compared to the contralateral side. To estimate the number of CD68^+^ cells in the thalamus, the object count of CD68^+^ cells was analyzed from 3–4 sagittal sections per hemisphere, taken every 500 µm between 1.9 and 3.4 mm or –1.9 and –3.4 mm relative to bregma, and the average cell count per hemisphere was used for further analysis.

### Quantitation of infarction size

At day 14 post-stroke, the average infarction size was quantified from 6 anti-NeuN–stained coronal brain sections, taken every 800 µm between 1.80 and –3.00 mm relative to bregma. The area devoid of NeuN^+^ cells and the total area of the brain section were defined in Pannoramic Viewer version 1.15.3. Infarction size is expressed as a percentage of the total area of the section. At day 2 post-stroke, the infarction volume was quantified with 2,3,5-triphenyltetrazolium chloride (TTC) staining from 2-mm brain slices as described previously (Airavaara et al., 2009).

### Measurement of TNF-α secretion from microglia/macrophages isolated from the stroke cortex

The CD11b immunopositive cortical microglia/macrophages were isolated by magnetic activated cell sorting (MACS). The rats were anesthetized with pentobarbital (90 mg/kg i.p.) at 7 d post-stroke after 90 min dMCAo and perfused with 200 ml saline. The ischemic cortex and the corresponding contralateral cortex were dissected on ice in HBSS without Ca^2+^ and Mg^2+^. The tissue was dissociated using Neural Tissue Dissociation kit (Miltenyi Biotec) and gentleMACS Dissociator (Miltenyi Biotec). After dissociation, the cells were suspended in 0.5% BSA in PBS and incubated with Myelin Removal Beads II (1:10, Miltenyi Biotec) for 15 min at 4°C. The cells were washed and resuspended in 0.5% BSA in PBS and filtered through an LS column (Miltenyi Biotec) using a QuadroMACS Separator (Miltenyi Biotec). The total effluent was collected and resuspended in 0.5% BSA with 2 mm EDTA in PBS. The cells were incubated at 4°C for 10 min with mouse anti-CD11b:FITC antibody (1:10, #MCA275FA, RRID:AB_2129486, AbD Serotec). The cells were washed, resuspended in 0.5% BSA with 2 mm EDTA in PBS, and incubated with anti-FITC MicroBeads (1:10, RRID:AB_244371, Miltenyi Biotec) for 15 min at 4°C. The cells were washed and resuspended in 0.5% BSA with 2 mm EDTA in PBS. The cell suspension was applied to an LS column placed on a QuadroMACS Separator, and the magnetically labeled cells were used for additional experiments. The cells were plated on poly-ornithine–coated glass coverslips on a 48-well plate at the density of 30,000 cells/well in DMEM:F12 medium (Life Technologies) containing 10% FBS and 0.2% primocin (InvivoGen). LPS or naloxone was added immediately after plating. The culture medium was collected 20 h later and analyzed using the Rat TNF alpha ELISA Ready-SET-Go! kit (#88-7340-22, RRID:AB_2575088, eBioscience). The results are presented from 3 independent experiments. The CD11b^+^ cells were isolated from 2–3 rats in each experiment. The values for each well within an experiment were normalized to the mean value of the control wells within the corresponding experiment.

### Experimental design and statistical analysis

All the experiments and analyses were performed in a blinded manner. First, the time course of glial activation in the dMCAo model was characterized. To minimize the number of animals needed (*n* = 24 in total; *n* = 4 per group), 90-min occlusion time was used to create a robust lesion. Immunostaining of sagittal sections with anti-Iba1, anti-CD68, and anti-GFAP antibodies was used to visualize the temporal and spatial distribution of glial cells post-stroke. As the dMCAo model is unilateral, the contralateral hemisphere was used as a control after we confirmed that the expression of glial cells in naive brain is similar to the contralateral hemisphere of ischemic brain. For quantitation of CD68^+^ cells, 3–4 sections per animal per hemisphere were used to result in an average.

Second, to test the efficacy of post-stroke (+)-naloxone (0.32 mg/kg; *n* = 27), repeated dosing (twice a day for 7 d) was used owing to the short half-life of naloxone. To make the setting clinically relevant, the treatment was started 1 d after stroke and was given intranasally, and a smaller infarct using 60-min dMCAo was induced. Behavioral recovery was monitored for 14 d with body asymmetry test, Bederson’s score, and measurement of spontaneous locomotor activity. Vehicle (*n* = 25) and no-treatment groups (*n* = 13) were used as a control in the behavioral assays. As both naloxone enantiomers have anti-inflammatory effects on microglia, only the (–) form antagonizes opioid receptors. Thus, we included also (–)-naloxone (*n* = 7) group in the behavioral assays to provide information about whether (+)- and (–)-naloxone have similar effects on recovery. The dose–response of (+)-naloxone was tested in a separate experiment (0.0008 mg/kg, *n* = 8; 0.008 mg/kg, *n* = 8; 0.08 mg/kg, *n* = 7; 0.8 mg/kg, *n* = 8; vehicle, *n* = 11). The amount of microglial activation in the striatum and thalamus and neuronal loss in the cortex and thalamus were analyzed with immunohistochemistry. For quantitation of Iba1^+^ and NeuN^+^ cells, 3–6 sections from corresponding coronal planes from each animal were used.

Statistical analyses were performed with IBM SPSS Statistics software version 24.0. Normal distribution of each dataset was analyzed by Levene’s test for equality of variances and analyzed with either one-way ANOVA or Kruskal–Wallis nonparametric ANOVA, or with Student’s *t* test or nonparametric Mann–Whitney *U* test in the case of only two groups. One-way ANOVA was followed by Bonferroni’s or Dunnett’s *post hoc* test and Kruskal–Wallis test was followed by pairwise comparison with Mann–Whitney *U* test. Since the immunohistochemical data did not differ between the vehicle and no treatment groups or between (+)-naloxone doses 0.32 and 0.8 mg/kg, the two groups were combined as one control group and one (+)-naloxone group. Statistical significance was considered at *p* < 0.05. The results are presented as mean ± SEM. Superscript letters listed with significance values correspond to the statistical tests shown in Table 1.

**Table 1.** Statistical analysis

Location	Dataset	Data structure	Type of test	Power
a	Fig. 2P	Non-normal distribution (unequal variances)	Kruskal–Wallis, Mann–Whitney *U*	*H(*11) = 41.5, *p* = 0.000; ipsilateral hemisphere: d2 vs. d7: *p* = 0.021; d2 vs. d14: *p* = 0.021; d2 vs. d28: *p* = 0.021; d2 vs. d56: *p* = 0.021; d2 vs. d112: *p* = 0.021; d7 vs. d14: *p* = 0.021; d7 vs. d28: *p* = 0.021; d7 vs. d56: *p* = 0.083; d7 vs. 112: *p* = 0.083; d14 vs. d28: *p* = 1.00; d14 vs. d56: *p* = 0.043; d14 vs. d112: *p* = 0.021; d28 vs. d56: *p* = 0.083; d28 vs. d112: *p* = 0.043; d56 s. d112: *p* = 0.773; ipsilateral vs. contralateral: d2: *p* = 0.773; d7: *p* = 0.021; d14: *p* = 0.021; d28: *p* = 0.021; d56: *p* = 0.021; d112: *p* = 0.021
b	Fig. 5B	Non-normal distribution	Kruskal–Wallis, Mann–Whitney *U*	d1: *H*(2) = 2.26, *p* = 0.323; d3: *H*(2) = 4.93, *p* = 0.085; d7: *H*(2) = 1.29, *p* = 0.524; d10: *H*(2) = 15.5, *p* = 0.000; NT vs. (+)-Nal: *p* = 0.006; Veh vs. (+)-Nal: *p* = 0.000; NT vs. Veh: *p* = 0.651; d14: *H*(2) = 12.3, *p* = 0.002; NT vs. (+)-Nal: *p* = 0.012; Veh vs. (+)-Nal: *p* = 0.001; NT vs. Veh: *p* = 0.485
c	Fig. 5C	Non-normal distribution	Kruskal–Wallis, Mann–Whitney *U*	d1: *H*(2) = 3.25, *p* = 0.197; d3: *H*(2) = 6.92, *p* = 0.032; NT vs. (+)-Nal: *p* = 0.017; Veh vs. (+)-Nal: *p* = 0.059; NT vs. Veh: *p* = 0.393; d7: *H*(2) = 3.26, *p* = 0.196; d10: *H*(2) = 15.4, *p* = 0.000; NT vs. (+)-Nal: *p* = 0.002; Veh vs. (+)-Nal: *p* = 0.002; NT vs. Veh: *p* = 0.203; d14: *H*(2) = 19.1, *p* = 0.000; NT vs. (+)-Nal: *p* = 0.000; Veh vs. (+)-Nal: *p* = 0.000; NT vs. Veh: *p* = 0.209
d	Fig. 5D	Non-normal distribution	Kruskal–Wallis, Mann–Whitney *U*	*H*(4) = 15.1, *p* = 0.004; Veh vs. 8 × 10^−4^: *p* = 0.206; Veh vs. 8 × 10^−3^: *p* = 0.966; Veh vs. 8 × 10^−2^: *p* = 0.002; Veh vs. 8 × 10^−1^: *p* = 0.059; 8 × 10^−4^ vs. 8 × 10^−3^: *p* = 0.552; 8 × 10^−4^ vs. 8 × 10^−2^: *p* = 0.001; 8 × 10^−4^ vs. 8 × 10^−1^: *p* = 0.019; 8 × 10^−3^ vs. 8 × 10^−2^: *p* = 0.031; 8 × 10^−3^ vs. 8 × 10^−1^: *p* = 0.142; 8 × 10^−2^ vs. 8 × 10^−1^: *p* = 0.488
e	Fig. 5E	Non-normal distribution	Kruskal–Wallis, Mann–Whitney *U*	*H*(4) = 6.38, *p* = 0.041; Veh vs. 8 × 10^−4^: *p* = 0.690; Veh vs. 8 × 10^−3^: *p* = 0.487; Veh vs. 8 × 10^−2^: *p* = 0.016; Veh vs. 8 × 10^−1^: *p* = 0.038; 8 × 10^−4^ vs. 8 × 10^−3^: *p* = 0.314; 8 × 10^−4^ vs. 8 × 10^−2^: *p* = 0.006; 8 × 10^−4^ vs. 8 × 10^−1^: *p* = 0.025; 8 × 10^−3^ vs. 8 × 10^−2^: *p* = 0.144; 8 × 10^−3^ vs. 8 × 10^−1^: *p* = 0.208; 8 × 10^−2^ vs. 8 × 10^−1^: *p* = 0.730
f	Fig. 5F	Normal distribution	One-way ANOVA, Bonferroni	*F*(2,42) = 0.054; NT vs. (+)-Nal: *p* = 0.064; Veh vs. (+)-Nal: *p* = 0.242; NT vs. Veh: *p* = 1.00
g	Fig. 5G	Non-normal distribution (unequal variances)	Kruskal–Wallis, Mann–Whitney *U*	*H*(2) = 6.82, *p* = 0.033; NT vs. (+)-Nal: *p* = 0.004; Veh vs. (+)-Nal: *p* = 0.243; NT vs. Veh: *p* = 0.313
h	Fig. 5H	Non-normal distribution	Mann–Whitney *U*	d1: *p* = 0.663; d3: *p* = 0.663; d7: *p* = 0.963; d10: *p* = 0.001; d14: *p* = 0.000
i	Fig. 5I	Non-normal distribution	Mann–Whitney *U*	d1: *p* = 0.159; d3: *p* = 0.401; d7: *p* = 0.565; d10: *p* = 0.002; d14: *p* = 0.005
j	Fig. 5J	Normal distribution	*t* test	d7: t(16) = 0.47, *p* = 0.647; d14: t(16) = 0.06, *p* = 0.953
k	Fig. 5K	Normal distribution	One-way ANOVA, Bonferroni	d7: *F*(2,59) = 5.27, *p* = 0.008; NT vs. (+)-Nal: *p* = 0.574; Veh vs. (+)-Nal: *p* = 0.113; NT vs. Veh: *p* = 0.009; d14: *F*(2,62) = 1.79, *p* = 0.175
l	Fig. 6A	Normal distribution	*t* test	t(26) = 2.51, *p* = 0.019
m	Fig. 6B	Non-normal distribution (unequal variances)	Kruskal–Wallis, Mann–Whitney *U*	*H*(2) = 11.4, *p* = 0.003; Naïve vs. Ctrl: *p* = 0.002; Naïve vs. (+)-Nal: *p* = 0.157; Ctrl vs. (+)-Nal: *p* = 0.036
n	Fig. 7A	Normal distribution	One-way ANOVA, Bonferroni	*F*(2,31) = 8.63, *p* = 0.001; Naïve vs. Ctrl: *p* = 0.003; Naïve vs. (+)-Nal: *p* = 1.00; Ctrl vs. (+)-Nal: *p* = 0.013
o	Fig. 7B	Non-normal distribution (unequal variances)	Kruskal–Wallis, Mann–Whitney *U*	*H*(2) = 5.77, *p* = 0.056
p	Fig. 7C	Non-normal distribution (unequal variances)	Kruskal–Wallis, Mann–Whitney *U*	*H*(2) = 17.6, *p* = 0.000; Naïve vs. Ctrl: *p* = 0.000; Naïve vs. (+)-Nal: *p* = 0.001; Ctrl vs. (+)-Nal: *p* = 0.027
q	Fig. 8B	Normal distribution	*t* test	t(13) = 0.89, *p* = 0.389
r	Fig. 8D	Non-normal distribution	Mann–Whitney *U*	d2: *p* = 0.861; d6: *p* = 0.825; d10: *p* = 0.279; d16: *p* = 0.066
s	Fig. 8F	Non-normal distribution	Mann–Whitney *U*	d1: *p* = 0.800; d3: *p* = 0.861; d7: *p* = 0.516; d10: *p* = 0.391; d14: *p* = 0.694
t	Fig. 8G	Normal distribution	Two-way RM ANOVA	*F*(4,52) = 0.29, *p* = 0.88
u	Fig. 9A	Non-normal distribution (unequal variances)	Mann–Whitney *U*	*p* = 0.006
v	Fig. 9B	Normal distribution	One-way ANOVA, Dunnett	*F*(6,54) = 2.74, *p* = 0.022; 20µM (–)-Nal: *p* = 0.669; 50µM (–)-Nal: *p* = 0.020; 100µM (–)-Nal: *p* = 0.218; 20µM (+)-Nal: *p* = 0.492; 50µM (+)-Nal: *p* = 0.637; 100µM (+)-Nal: *p* = 0.006

(+)-Nal, (+)-naloxone; (–)-Nal, (–)-naloxone; Ctrl, control; d, post-stroke day; NT, no treatment; RM, repeated measures; Veh, vehicle.

## Results

### Long-lasting microglial and astrocyte activation in the ipsilateral hemisphere after dMCAo

**Figure 2.**
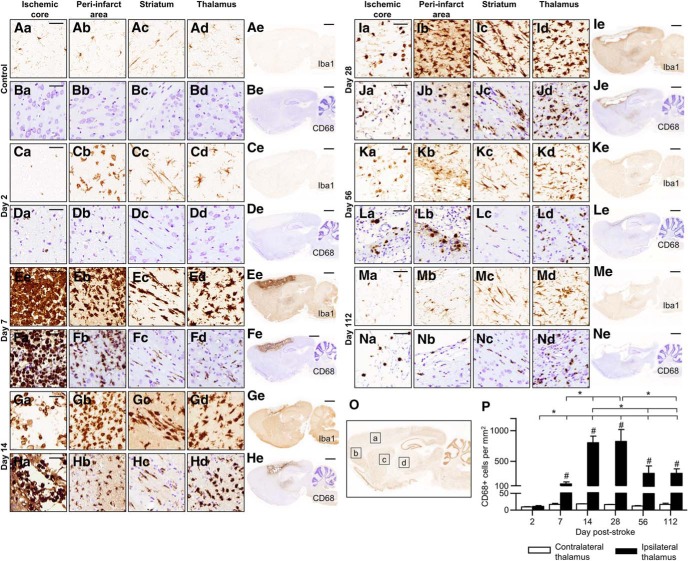
**Time course of microglia/macrophage activation after cortical stroke.** Representative images of immunostaining for all microglia/macrophages (Iba1) and phagocytic microglia/macrophages (CD68) from ischemic core (***a***), peri-infarct area (***b***), striatum (***c***), thalamus (***d***), and whole brain (***e***) sagittal sections at 2 (***C***, ***D***), 7 (***E***, ***F***), 14 (***G***, ***H***), 28 (***I***, ***J***), 56 (***K***, ***L***), and 112 (***M***, ***N***) days after 90-min dMCAO in rat. Control images (***A***, ***B***) are from the contralateral hemisphere of the stroke brain. Scale bar is 50 µm (high magnification) and 2000 µm (low magnification). ***O***, Example image of anti-NeuN immunostaining at post-stroke day 2 showing in more detail the regions ***a–d***. ***P***, Quantitation of CD68^+^ cells in the thalamus (***d***) at different time points showing the accumulation and clearance of phagocytic microglia/macrophages in the ipsilateral thalamus. *, *p* < 0.05 indicates statistical difference between the ipsilateral thalamus at different time points; #, *p* < 0.05 indicates statistical difference between the ipsilateral and contralateral thalamus in each time point, Mann–Whitney *U* test after Kruskal–Wallis test; *n* = 4 in each group. The data represent mean ± SEM.

**Figure 3.**
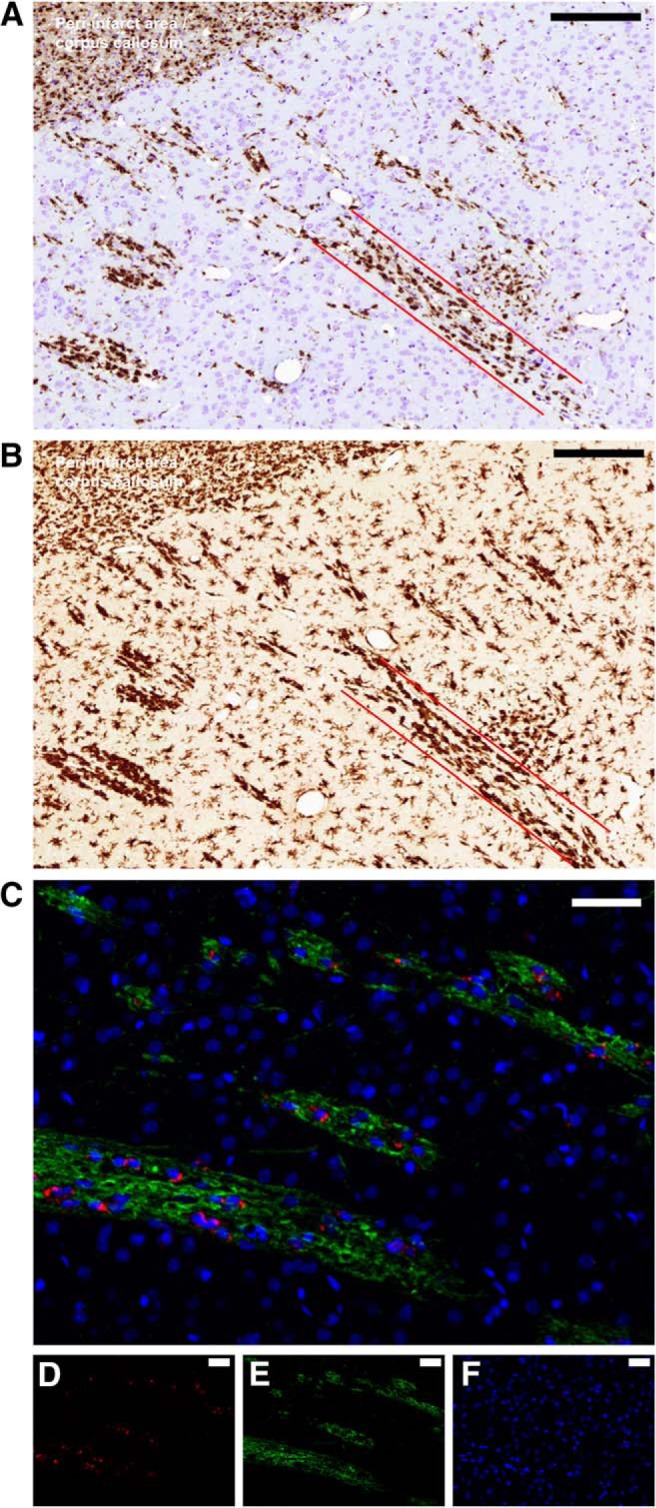
**Activated microglia in the striatum are lined up along axonal bundles after cortical stroke.** Immunostaining of rat striatum at post-stroke day 14 for phagocytic microglia/macrophages (CD68; ***A***) and all microglia/macrophages (Iba1; ***B***). **C–F**, Double immunofluorescence staining of rat striatum at post-stroke day 14 for phagocytic microglia/macrophages (CD68; red; ***D***) and myelin (MBP; green; ***E***) with DAPI (blue; ***F***). In ***A***, ***B***, scale bar is 200 µm; in ***C–F***, scale bar is 50 µm.

**Figure 4.**
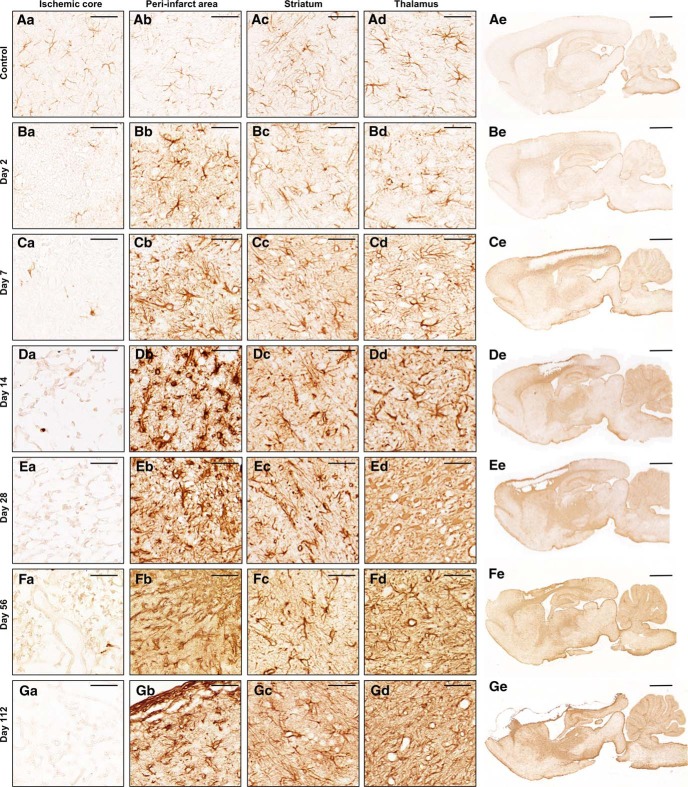
**Time course of astrocyte activation after cortical stroke.** Representative images of immunostaining for astrocytes (GFAP) from the ischemic core (***a***), peri-infarct area (***b***), striatum (***c***), and thalamus (***d***) at 2 (***B***), 7 (***C***), 14 (***D***), 28 (***E***), 56 (***F***), and 112 (***G***) days after 90-min dMCAo in sagittal rat brain paraffin sections. Control images (***A***) are from the contralateral hemisphere of the stroke brain. Scale bar is 50 µm in high-magnification images and 2000 µm in low-magnification images. The regions analyzed (***a–d***) are shown in more detail in Fig. 2*O*.

We first characterized the neuroinflammatory response in proximal (cortex) and distal (thalamus) regions after transient 90-min dMCAo, and second, tested the efficacy of post-stroke intranasal naloxone treatment in adult rats after 60 min dMCAo. At day 2 post-stroke, microglia/macrophage activation was observed mainly in the peri-infarct region, and few phagocytic CD68^+^ cells (a marker for activated, phagocytic microglia/macrophages) were present (Fig. 2*C*, *D*). Microglia/macrophage activation peaked on day 7 post-stroke in the ischemic cortex, when the core area was filled with Iba1^+^ cells (a marker for all microglia/macrophages) and CD68^+^ cells having a phagocytic/macrophage-type morphology (Fig. 2*Ea*, *Fa*). Microglia/macrophage activation was seen in the ipsilateral striatum starting from post-stroke day 7 (Fig. 2*Ec*). Interestingly, activated microglia/macrophages were aligned along fiber bundles, with a “stream-like” formation along the myelin embedded axons (Fig. 3). Iba1^+^ cells with activated morphology were evident in the ipsilateral thalamus at day 7 post-stroke, but only a few cells were CD68^+^ (Fig. 2*Ed*, *Fd*, *P*). CD68 immunoreactivity increased in the ipsilateral thalamus after post-stroke day 7, peaked at days 14–28, and remained elevated until day 112 (*H*(11) = 41.5, *p* < 0.001,^a^ Kruskal–Wallis, Fig. 2*P*). These results suggest that inflammation in the thalamus is long lasting and phagocytic cells are present in far distal areas at least 4 mo after stroke (Fig. 2*Nd*, *P*). Neuronal loss was observed in the ipsilateral thalamus at post-stroke day 14 (Fig. 6*B*). At 112 d post-stroke, atrophy of the thalamus due to shrinkage of the ipsilateral thalamus was observed (Fig. 2*Me*, *Ne*; Fig. 4*Ge*). Analysis of spatiotemporal activation of GFAP^+^ cells (a marker for astrocytes) revealed a pattern similar to that of microglia/macrophage activation (Fig. 4). Similarly to microglia, astrocytes in the ischemic core died during the first couple of days. Astrogliosis was found in the peri-infarct area starting from day 2 post-stroke (Fig. 4*Bb*). There was clear astrocytic scar formation in the peri-infarct region starting from post-stroke day 7 (Fig. 4*Cb*, *Ce*). From day 7 onward, astrogliosis was evident in the striatum and even in the thalamus (Fig. 4*C–G*). Astrogliosis in the ipsilateral thalamus persisted for up to 112 d post-stroke (Fig. 4*Gd*, *Ge*), suggesting glial scar formation without local ischemic damage.

### Post-stroke treatment with intranasal (+)-naloxone started on day 1 post-stroke promotes short-term behavioral recovery, reduces neuronal loss, and decreases microglial activation

**Figure 5.**
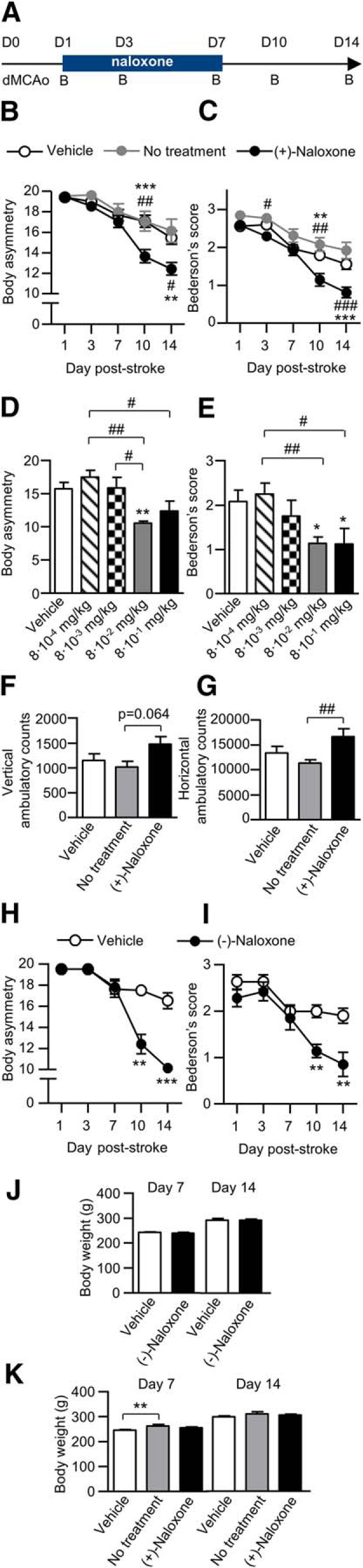
**Post-stroke intranasal administration of naloxone enantiomers promotes functional recovery. *A***, Experimental timeline. Intranasal naloxone (or vehicle) was administered twice daily for 7 d post-stroke. D1–D14, post-stroke days 1–14; B, behavioral assay. **B**, **C**, Effects of (+)-naloxone (0.32 mg/kg; *n* = 27), vehicle (*n* = 25), and no treatment (*n* = 13) on body asymmetry (***B***) and Bederson’s neurologic score test (***C***). **, *p* < 0.01 and ***, *p* < 0.001 indicate *post hoc* comparison between (+)-naloxone and vehicle groups, and #, *p* < 0.05, ##, *p* < 0.01, and ###, *p* < 0.001 indicate *post hoc* analysis between (+)-naloxone and no-treatment groups with Mann–Whitney *U* test after Kruskal–Wallis test. **D**, **E**, Effects of different doses of (+)-naloxone, 0.0008 mg/kg (*n* = 8), 0.008 mg/kg (*n* = 8), 0.08 mg/kg (*n* = 7), and 0.8 mg/kg (*n* = 8), compared to vehicle (*n* = 11) on day 14 post-stroke on body asymmetry (***D***) and Bederson’s neurologic score test (***E***). *, *p* < 0.05 and **, *p* < 0.01 indicate pairwise comparison with vehicle; #, *p* < 0.05 and ##, *p* < 0.01 indicate pairwise comparison with other (+)-naloxone doses with Mann–Whitney *U* test after Kruskal–Wallis test. **F**, **G**, Effects of (+)-naloxone (0.32 mg/kg; *n* = 16), vehicle (*n* = 16), and no treatment (*n* = 13) on vertical (***F***) and horizontal (***G***) activity measured for 24 h on day 14. ##, *p* < 0.01, Mann–Whitney *U* test after Kruskal–Wallis test. ***H–J***, Effects of (–)-naloxone (0.32 mg/kg; *n* = 7) and vehicle (*n* = 11) on body asymmetry test (***H***), Bederson’s neurologic score test (***I***), and body weight (***J***). **, *p* < 0.01 and ***, *p* < 0.001 indicate comparison with vehicle group with Mann–Whitney *U* test. ***K***, Effects of (+)-naloxone (0.32 mg/kg, *n* = 27), vehicle (*n* = 25), and no treatment (*n* = 13) on body weight on days 7 and 14 post-stroke. **, *p* < 0.01, one-way ANOVA, Bonferroni’s *post hoc* test. The data represent mean ± SEM.

The efficacy of post-stroke (+)-naloxone treatment was tested in rats (*n* = 65), and treatment was initiated 1 d after 60-min dMCAo, before extensive activation of microglia/macrophages, and it was continued for 7 d, i.e., the period when microglia/macrophage activation progresses in the ischemic region. One day post-stroke, the rats were balanced into groups based on their neurologic deficits as measured by body asymmetry and Bederson’s score, and (+)-naloxone (0.32 mg/kg) or vehicle was administered intranasally every 12 h for 7 d (i.e., 14 doses per animal, Fig. 5*A*). To identify the confounding effect of isoflurane, two control groups were included: (i) stroke rats receiving intranasal vehicle (including repeated isoflurane) and (ii) stroke rats with no intranasal treatment and no isoflurane. There were no significant differences in body asymmetry among the groups on days 1, 3, or 7 after dMCAo^b^ (Fig. 5*B*). However, on days 10 and 14 post-stroke, body asymmetry (*H*(2) = 15.5, *p* < 0.001 and *H*(2) = 12.3, *p* = 0.002, respectively,^b^ Kruskal–Wallis) and neurologic deficits (*H*(2) = 15.4, *p* < 0.001 and *H*(2) = 19.1, *p* < 0.001, respectively^c^) were significantly reduced in the (+)-naloxone treated rats (Fig. 5*B*, *C*). The reduction in body asymmetry (*H*(4) = 15.1, *p* = 0.004,^d^ Kruskal–Wallis) and neurologic deficits (*H*(4) = 6.38, *p* = 0.041^e^) was dose dependent (Fig. 5*D*, *E*). As another indicator of hastened recovery, (+)-naloxone improved locomotor activity at day 14 post-stroke (*H*(2) = 6.82, *p* = 0.033,^g^ Kruskal–Wallis, Fig. 5*G*). Similarly, (–)-naloxone (0.32 mg/kg, intranasally) induced behavioral recovery on days 10 and 14 post-stroke (*n* = 18; *p* = 0.001 and *p* < 0.001, respectively,^h^ for body asymmetry test; and *p* = 0.002 and *p* = 0.005, respectively,^i^ for Bederson’s score, Mann–Whitney *U* test; Fig. 5*H*, *I*). It has been reported that continuous administration of the opioid receptor antagonist (–)-naltrexone decreases body weight and appetite (Atkinson, 1984). We found no effect on body weight with naloxone treatment^j,k^ (Fig. 5*J*, *K*).

**Figure 6.**
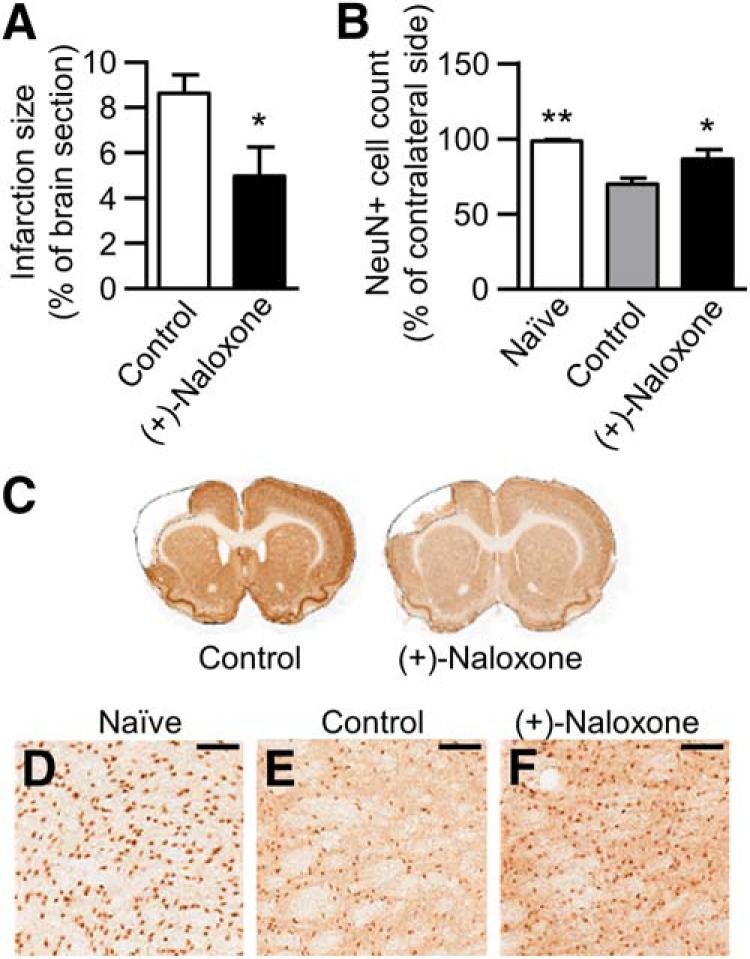
**Post-stroke intranasal (+)-naloxone decreases infarction area and neuronal loss in the thalamus. *A***, Average infarction size calculated from NeuN-negative area at day 14 post-stroke. *, *p* < 0.05, Student’s *t* test. ***B***, Average number of neurons (NeuN^+^ cells) in the ipsilateral thalamus at day 14 post-stroke expressed as a percentage of the contralateral thalamus. *, *p* < 0.05 and **, *p* < 0.01 indicate pairwise comparison with the control group with Mann–Whitney *U* test following Kruskal–Wallis test. ***C***, Representative photomicrographs of anti-NeuN immunostained brain sections, with infarction area delineated. ***D–F***, Representative photomicrographs of anti-NeuN immunostaining of ipsilateral thalamus in naive (***D***), control (***E***), and (+)-naloxone–treated (***F***) rats. Scale bar is 150 µm. Naive, no-stroke rats (*n* = 6); control, stroke rats with vehicle or no treatment (*n* = 18); (+)-naloxone, 0.32–0.8 mg/kg (*n* = 10). The data represent mean ± SEM.

**Figure 7.**
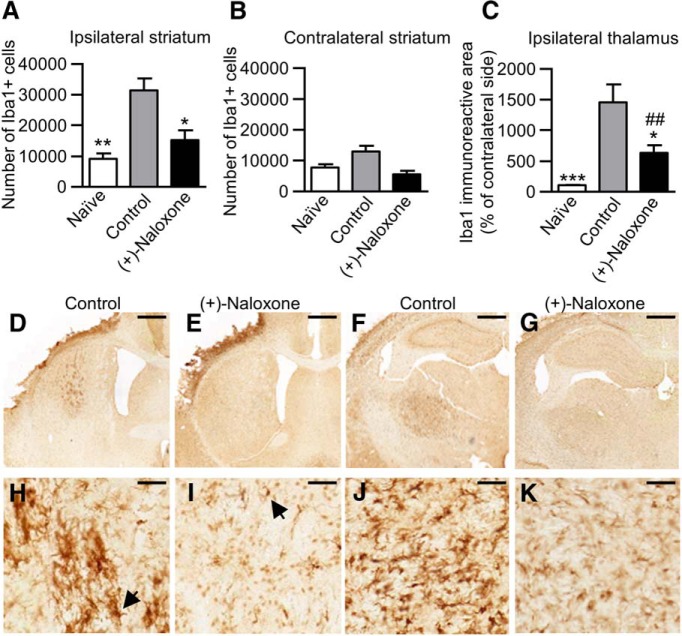
**Post-stroke intranasal (+)-naloxone decreases microglia/macrophage activation in the striatum and thalamus. *A***, ***B***, Microglia/macrophages (Iba1^+^ cells) were counted with unbiased stereology in the ipsilateral (***A***) and contralateral (***B***) striatum. *, *p* < 0.05 and **, *p* < 0.01 indicate pairwise comparison with the control group with Bonferroni’s *post hoc* test following one-way ANOVA. ***C***, The area of Iba1^+^ cells in the ipsilateral thalamus expressed as a percentage of the contralateral thalamus. *, *p* < 0.05 and ***, *p* < 0.001 indicate pairwise comparison with the control group; ##, *p* < 0.01 indicates comparison with the naive group with Mann–Whitney *U* test following Kruskal–Wallis test. ***D–K***, Representative photomicrographs of anti-Iba1 immunostaining of ipsilateral striatum (***D***, ***E***) and thalamus (***F***, ***G***) in control (***D***, ***F***) and (+)-naloxone–treated (***E***, ***G***) rats; ***H–K*** show high magnification. Black arrow shows a typical Iba1^+^ cell. Scale bars are 1000 µm (***D–G***) and 50 µm (***H–K***). Naive, no-stroke rats (*n* = 6); control, stroke rats with vehicle or no treatment (*n* = 18); (+)-naloxone, 0.32–0.8 mg/kg (*n* = 10). The data represent mean ± SEM.

Post-stroke (+)-naloxone (0.32–0.8 mg/kg) reduced infarction size on day 14 after dMCAo (*t*(26) = 2.51, *p* = 0.019,^l^ unpaired *t* test; Fig. 6*A*) and prevented delayed neuronal death in the ipsilateral thalamus (*H*(2) = 11.4, *p* = 0.003,^m^ Kruskal–Wallis; Fig. 6*B*). We observed a negative correlation between the number of neurons (NeuN^+^ cells) in the ipsilateral thalamus and infarction size (Pearson correlation *R* = –0.691, *p* < 0.0001), showing that the larger the lesion in the cortex, the more extensive neuronal loss in the thalamus. (+)-Naloxone significantly decreased the Iba1^+^ cell number in the ipsilateral striatum at post-stroke day 14 (*F*(2,31) = 8.63, *p* = 0.001,^n^ one-way ANOVA; Fig. 7*A*). There was no statistical difference in the contralateral striatum^o^ (Fig. 7*B*). Similarly, a significant reduction of Iba1 immunoreactivity by (+)-naloxone was found in the ipsilateral thalamus (*H*(2) = 17.6, *p* < 0.001,^p^ Kruskal–Wallis; Fig. 7*C*).

### Pretreatment or delayed post-stroke treatment with (+)-naloxone is not beneficial in dMCAo model

**Figure 8.**
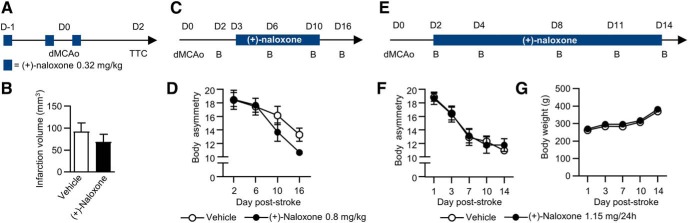
**Effect of different pre- and post-stroke treatment with (+)-naloxone on infarct volume and functional recovery. *A***, Experimental timeline. Intranasal (+)-naloxone or vehicle was administered three times: 12 and 1 h before dMCAo and immediately after reperfusion. Infarction volume was determined by TTC staining 2 d after stroke. ***B***, Average infarction volume (mm^3^) on day 2 post-stroke in vehicle (*n* = 7) and (+)-naloxone (0.32 mg/kg; *n* = 8) pretreated rats. Prestroke intranasal administration of (+)-naloxone was not neuroprotective in 60-min dMCAo. ***C***, Experimental timeline. (+)-Naloxone was delivered intranasally twice daily for 7 d post-stroke starting from post-stroke day 3. ***D***, The effect of (+)-naloxone (0.8 mg/kg; *n* = 6) and vehicle (*n* = 7) treatment from post-stroke day 3 to post-stroke day 10 on body asymmetry test. ***E***, Experimental timeline. (+)-Naloxone was delivered into the ventricle via mini-osmotic pumps for 12 d post-stroke starting from post-stroke day 2. ***F***, ***G***, The effects of 12-d continuous delivery of (+)-naloxone (1.15 mg/24 h; *n* = 7) and vehicle (*n* = 8) on body asymmetry test (***F***) and body weight (***G***). In ***A***, ***C***, and ***E***: D, indicated post-stroke day; B, behavioral assay. The data represent mean ± SEM.

We tested the neuroprotective effect of (+)-naloxone (*n* = 15) by giving (+)-naloxone (0.32 mg/kg) or vehicle intranasally three times: 12 and 1 h before 60-min dMCAo and immediately after reperfusion (Fig. 8*A*). The infarction volume was determined by TTC staining 2 d after stroke. We found no significant differences in the infarction volume between the groups (*p* = 0.389,^q^ unpaired *t* test; Fig. 8*B*). To find out whether more delayed (+)-naloxone treatment would give similar effect on recovery as the treatment started on post-stroke day 1, we administered (+)-naloxone (0.8 mg/kg) or vehicle intranasally twice daily starting from day 3 after 60-min dMCAo and continuing for 7 d (*n* = 13; Fig. 8*C*). There was an evident recovery effect over time in the body asymmetry test, but there were no statistically significant differences between the vehicle and (+)-naloxone groups^r^ (Fig. 8*D*). On post-stroke day 16, there was a tendency (*p* = 0.066,^r^ Mann–Whitney *U* test) in the (+)-naloxone group for milder neurologic deficits in the body asymmetry test, but it did not reach statistical significance. To study whether longer, continuous administration of (+)-naloxone would further enhance recovery after 60-min dMCAo, we delivered (+)-naloxone (1.15 mg/24 h) or vehicle into the ventricle using mini-osmotic pumps on post-stroke days 2–14 (*n* = 15; Fig. 8*E*). There was again evident recovery effect in the body asymmetry test, but there were no differences between the vehicle and (+)-naloxone groups^s^ (Fig. 8*F*), nor were there any differences in body weight between the groups^t^ (Fig. 8*G*).

### Naloxone decreases TNF-α secretion from microglia/macrophages

**Figure 9.**
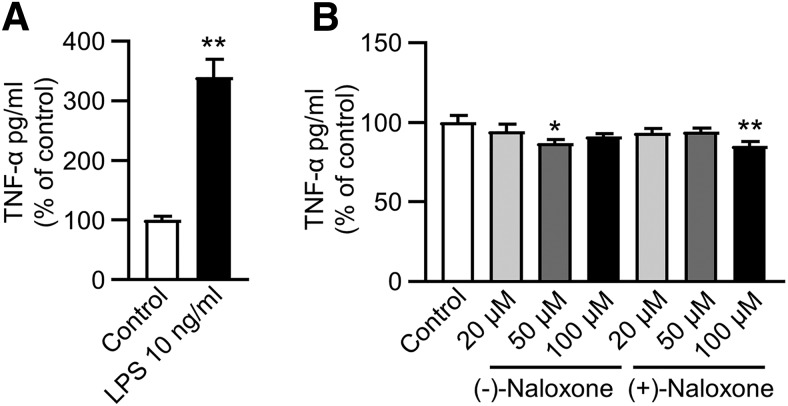
**Naloxone reduced TNF-α secretion from CD11b^+^ cells. *A***, LPS induced TNF-α secretion from CD11b-expressing cells isolated from the infarct area of rat brain 7 d after dMCAo. **, *p* < 0.01, Mann–Whitney *U* test; control *n* = 6, LPS *n* = 5 in 2 independent experiments. ***B***, CD11b^+^ cells were isolated from the infarct area and treated with different concentrations of naloxone as indicated for 20 h. *, *p* < 0.05 and **, *p* < 0.01 indicate pairwise comparison with the control group by Dunnett’s *post hoc* test following one-way ANOVA; control *n* = 9, naloxone *n* = 8–9 in 3 independent experiments. The culture medium was analyzed using TNF-α ELISA. The data represent mean ± SEM.

To test whether naloxone enantiomers affect cytokine secretion, we isolated CD11b^+^ microglia/macrophages from the infarct area at day 7 post-stroke and measured the secretion of TNF-α, a cytokine with well-characterized pro-inflammatory effects downstream of TLR4 signaling. First, we tested whether the isolated cells respond to treatment with LPS by increasing the secretion of TNF-α. Overnight treatment with LPS increased the amount of secreted TNF-α ∼3.5-fold (*p* = 0.006,^u^ Mann–Whitney *U*; Fig. 9*A*), confirming that the isolated cell population had properties characteristic of microglia and macrophages. Overnight treatment with both naloxone enantiomers significantly decreased the amount of TNF-α in the culture medium (*F*(6,54) = 2.74, *p* = 0.022,^v^ one-way ANOVA; Fig. 9*B*). (–)-Naloxone 50 µM and (+)-naloxone 100 µM decreased the unstimulated secretion of TNF-α by ∼15% compared with control.

## Discussion

We show for the first time that intranasal post-stroke administration of naloxone enantiomers reduces inflammation and hastens recovery during short-term behavioral monitoring. Our data support a therapeutic window for initiation of twice daily (+)-naloxone treatment between 16 and 36 h post-stroke and continuing the treatment for 7 d. Our study also indicates that modulation of microglia/macrophage activation in the ischemic cerebral cortex and remote regions in the striatum and thalamus is a potential therapeutic drug strategy. Because microglia/macrophages can phagocytose viable neurons after ischemia (Neher et al., 2013), neuroinflammation may lead to secondary neuronal loss. An ischemic brain injury leads to activation of microglia and the release of pro-inflammatory factors and further potentiates neuronal damage days to weeks after dMCAo (Dirnagl et al., 1999). It has been suggested that during an ischemic event, the brain remains in a continual state of neurotoxicity and microglia become overactivated, releasing pro-inflammatory factors that can contribute to further damage (Glass et al., 2010). It is known that overreactive microglia can cause increased levels of cytokines, specifically TNF-α and interleukin-1β and -6 (Lee et al., 1993; Block et al., 2007). Therefore anti-inflammatory drugs, such as minocycline, have been used to alleviate this neuroinflammation and improve stroke outcome (Yrjanheikki et al., 1999). Liebigt et al. (2012) have shown that post-stroke application of minocycline and indomethacin in rats, combined with rehabilitative training, produces improved functional recovery compared with training alone.

Our data indicate that twice-a-day post-stroke treatment with (+)-naloxone for a week improves short-term behavioral recovery and reduces neuronal damage and infarction size. The behavioral effect of (+)-naloxone correlated with suppression of activated microglia in the striatum and thalamus and was not observed until day 10 post-stroke, which suggests that (+)-naloxone targets delayed post-stroke pathophysiological mechanisms such as inflammation. The administration of (+)-naloxone was targeted to the period when the number of activated microglia/macrophages is peaking in the cerebral cortex and before microglial activation is evident in the striatum or thalamus. We therefore propose that dampening microglia/macrophage activation with post-stroke (+)-naloxone restricts inflammation to limit associated lesion expansion that occurs up to 24–48 h after transient MCAo (Li et al., 2000; Liu et al., 2009). This is further supported by the fact that (+)-naloxone treatment starting on days 2–3 post-stroke did not have a significant effect on recovery. Thus, the therapeutic window for (+)-naloxone seems to be between 16 and 36 h post-stroke, when there is not yet substantial activation of microglia. However, when using mini-osmotic pumps, the drug kinetics and concentrations at the target tissues over the administration period are only estimations until quantitative assessments of delivery are performed. When the intranasal (+)-naloxone treatment was started at day 3 post-stroke and continued for 7 d, there was a trend for milder neurologic deficits on day 16 post-stroke (*p* = 0.066). Because of the small number of animals, the data are inconclusive and further studies are needed to fully optimize the dosing and timing of administration. Yet, prestroke treatment with (+)-naloxone was not neuroprotective, as has been shown before by Liao et al. (2003), supporting our hypothesis that (+)-naloxone should be administered during the inflammation period.

We also show that naloxone enantiomers decrease unstimulated TNF-α secretion from microglia/macrophages isolated from the stroke cortex at day 7 post-stroke. Although the characteristics and phenotypes of microglia are altered in *in vitro* conditions (Gosselin et al., 2017), these data strengthen the view that (+)-naloxone can regulate the pro-inflammatory function of microglia/macrophages. Moreover, microglia phenotype is highly dependent on the environment where it is, and therefore the data should be interpreted carefully. It is noteworthy that this study is the first to show that (–)- or (+)-naloxone inhibits basal TNF-α secretion from stroke-activated microglia/macrophages without extra stimulus.

The time course of microglial and astrocyte activation in the thalamus after cortical stroke is less studied. We observed activated microglia/macrophages and increased GFAP immunoreactivity in the thalamus still at 4 mo post-stroke, and indeed, also in the intraluminal MCAo model microglial activation in the thalamus has been reported to be long-lasting, for up to 6 mo post-stroke (Justicia et al., 2008). However, in the intraluminal MCAo model, the ischemic lesion is somewhat close to the thalamus and in some cases extends into it. Microglia and astrocytes have been shown to be activated already after day 3 post-stroke in the ipsilateral thalamus in the transient intraluminal MCAo model (Loos et al., 2003). In our model, microglial and astrocyte activation was evident in the ipsilateral thalamus at 7 d post-stroke, but interestingly, microglia were not phagocytic until day 14. Previously, it has been reported that neuronal degeneration in the ipsilateral thalamus is evident at 14 d post-stroke in the transient intraluminal MCAo model (Loos et al., 2003), similar to our findings. The ischemia-induced neuronal loss and microglia/macrophage activation in the thalamus probably reflects retrograde/anterograde degeneration caused by cortical damage. However, inflammation has been implicated in secondary neurodegeneration (Block et al., 2005; Schroeter et al., 2006), and (+)-naloxone reduces inflammation and neurodegeneration at both primary (cortex) and secondary (thalamus) sites of injury. Although it remains unclear whether the beneficial effects of (+)-naloxone occur directly in cells in the thalamus or indirectly by reducing inflammation in the cortex, or both, intranasal (+)-naloxone is apparently beneficial to recovery from stroke. The thalamus regulates multiple sensory and motor functions that are also controlled by other brain regions. Furthermore, the thalamus is a relay station connecting the right and left hemispheres, and a unilateral lesion of the thalamus usually has little behavioral consequences (Carrera and Bogousslavsky, 2006). Overall, the role of thalamic neurodegeneration and inflammation following cortical infarction remains unclear, and it is not known whether the secondary pathology affects behavioral recovery. Our study supports the need for future research into the role of thalamic injury in cortical stroke.

Single (+)-naloxone doses that had effect on recovery in our study were 0.08 mg/kg, 0.32 mg/kg, and 0.8 mg/kg. As a comparison, the FDA-approved Narcan nasal spray to treat opioid overdose contains 4 mg of (–)-naloxone hydrochloride, being equivalent to 0.08 mg/kg dose for a person weighing 50 kg. Based on pharmacokinetic data on naloxone (Dowling et al., 2008; Krieter et al., 2016), we estimated that (+)-naloxone levels in rat brain reached nanomolar concentrations. The pharmacokinetic profile of naloxone is favorable for stroke treatment, since it is efficiently transported to the brain. However, its short elimination half-life [1.57 ± 0.784 h (Lewis et al., 2012)] requires repeated dosing. Short-term (up to 2 d post-stroke) intravenous (–)-naloxone (dose from 0.4 to 4 mg) has been tested in patients having an acute ischemic stroke, and it has rapidly, within minutes, improved neurologic deficits (Baskin and Hosobuchi, 1981; Jabaily and Davis, 1984). However, later clinical studies on the efficacy of naloxone have been inconclusive. A phase II open trial studying the safety of a high loading dose followed by 24-h infusion of (–)-naloxone did not find benefit at 3 mo post-stroke when the treatment was started within 48 h of stroke onset (Olinger et al., 1990). A subsequent double-blind, randomized pilot trial using the 24-h infusion found no significant differences between the naloxone and placebo groups when the treatment was initiated within 12 h of the onset of symptoms (Federico et al., 1991). At the time when these studies were performed, the anti-inflammatory effects of naloxone were not known. The lack of effect in these clinical studies may have resulted from the short treatment regimen. Treatment for only 24 h may not be enough to dampen the post-stroke microglial response. Moreover, the clinical studies have been based on the assumption that opioid antagonism is at least partly behind the beneficial effect of naloxone in acute stroke that was reported in the early case studies in the 1980s. In our experiment, both (+)- and (–)-naloxone had a similar effect on recovery starting from post-stroke day 10 onward, implying that opioid receptor antagonism is not necessary for the recovery promoting effect in the chronic phase of stroke. Thus, we propose additional clinical studies that would use different dosing paradigms to optimize the post-stroke (+)-naloxone dosing regimen.

The Stroke Therapy Academic Industry Roundtable (STAIR) recommendations to improve the quality of preclinical stroke studies emphasize the importance of dose–response and therapeutic window studies together with histologic and functional outcome monitoring. According to STAIR recommendations, the therapies should be tested in several animal species using both sexes as well as aged and comorbid animals. Therefore, we warrant further studies using (+)-naloxone for stroke treatment in female, aged, and comorbid animals to better reflect the clinical situation within the heterogeneous patient population. Also, behavioral testing should be conducted for >14 d to confirm the beneficial effect of (+)-naloxone on long-term behavioral recovery. Regarding the safety of naloxone therapy, naloxone has been already shown to be safe and well tolerated in patients with the corresponding dose range we have been using in rats.

In conclusion, characterization of the neuroinflammatory response in rat cortical stroke revealed long-lasting microglia/macrophage and astrocyte activation as well as neuronal death in the ipsilateral thalamus. Phagocytic cells were present in the thalamus for up to 4 mo post-stroke. This delayed neuronal loss and phagocytosis in the thalamus could serve as a new target for drug treatment after stroke with a larger therapeutic window than exists for current post-stroke treatment (i.e., tissue plasminogen activator). Most importantly, we found that intermittent post-stroke intranasal (+)-naloxone treatment starting on day 1 post-stroke promoted short-term behavioral recovery, reduced microglial activation in the striatum and thalamus, and decreased neuronal loss in the cortex and thalamus. It is likely that (+)-naloxone mediates its positive effects on stroke via mechanisms where TLR signaling and reduction of oxidative stress are involved. (+)-Naloxone is thus a promising drug for the treatment of ischemic stroke.

## Synthesis

Reviewing Editor: Chay Kuo, Duke University Decisions are customarily a result of the Reviewing Editor and the peer reviewers coming together and discussing their recommendations until a consensus is reached. When revisions are invited, a fact-based synthesis statement explaining their decision and outlining what is needed to prepare a revision will be listed below. The following reviewer(s) agreed to reveal their identity: Courtney DeVries, S Thomas Carmichael

Reviewer #1

This is an interesting manuscript which demonstrates in rats that intranasal naloxone is effective in reducing infarct size and improving behavioral recovery after dMCAO. Although none of the various aspects of the study are particularly novel (naloxone effects on stroke have been reported, intranasal naloxone administration has been used in both humans and rodents, and naloxone has been shown to suppress microglial activation and TNF in other inflammatory contexts), the use of intranasal naloxone to suppress stroke-induced microglial activation, which presumably then improves stroke outcome, is innovative. In addition, the demonstration of persistent microglial activation in the thalamus that lasts months after a relatively short MCA occlusion (1 h) is valuable new information. Furthermore, the current approach could be clinically valuable because it allows noninvasive central administration and remains effective when administered 24 h after cerebral ischemia. The main criticism of the study is that the behavioral analyses are rudimentary, and therefore not particularly convincing in terms of demonstrating long-term functional benefit, however, they are encouraging, and likely to prompt an extensive behavioral characterization.

Additional suggestions for manuscript improvement are as follows:

Line 74-76: “Our results are the first to show that by targeting microglia/macrophage activation with post-stroke (+) naloxone administration, functional recovery can be improved.” This statement should be tempered, although likely, this study does not demonstrate conclusively that it is naloxone's effects on microglia that are responsible for the functional recovery.

Line 360-361: “At post-stroke day one, the rats were balanced into groups based on their neurological deficits as measured by body asymmetry and Bederson's score...” What was the rationale for this non-random assignment to experimental groups? This is a particular concern given the small n/group. Were the behavioral assessments conducted by someone who was not aware of group assignment? This should be explicitly stated in the methods section. In addition, it should be stated how soon after drug treatment the behavioral tests were conducted.

Line 459-461: “However, when using mini osmotic pumps it always remains uncertain how efficient the continuous delivery of the drug is into the brain.” It is not clear what is meant by this statement- are the authors concerned about the accuracy of the pumping rates? A citation should be provided to support this statement.

The figure legends of bar graphs should indicate what the bars represent (presumably mean) and whether the error bars represent SEM or SD.

Figure 6B. The table reports significant differences between groups, but they are not indicated in the figure.

Figure 7D. The conclusion that there is no difference in behavior score is not particularly compelling, and may reflect low power. In addition, if there is truly no naloxone effect, it is not possible to tell if it is because the treatment was started one day too late, or because behavior testing occurred on day 16, rather than day 14 as in the other experiments. Were microglial measures taken for these animals? If the argument is that naloxone improves stroke outcome by suppressing microglia, then the microglial analyses should be provided for these studies too.

Reviewer #2

Antilla and colleagues report the use of an opioid antagonist, Naloxone, already in clinical use as a potential therapeutic for stroke recovery. The authors show that Naloxene dampens microglial activation and renders neuroprotection only when administered one day after a stroke, implying the existence of a therapeutic window. Although the use of naloxone in stroke is not a novel finding, these studies shed light on some of the pathophysiological changes induced by naloxone treatment and establish a time course that addresses past failure in the field. The dose response studies are also a strength.

The concerns in this paper are modest, as this provides important information for the field.

1. It is interesting to note that there is loss of the prefrontal cortex at latter time points implying continual stroke damage even 4 months after a stroke, unlike other stroke models. The loss could be attributed to secondary neuronal damage from neuroinflammation that is prevalent at these time points.

2. The authors imply that dampening microglial activation through naloxone treatment is what causes recovery (as in the Abstract). Based on the evidence presented, a reduction in microglial activation is likely the result of naloxone treatment but not necessarily what causes recovery. The authors could consider re-phrasing this.

3. 60-90 minutes is mentioned as duration of CCA occlusion. It appears that the 90 minute occlusion was used for the tissue analysis of microglia/macrophage markers and GFAP, and the behavioral testing was done on 60 minute bilateral CCA occlusion. The start of the Results section should clarify this.

4. Stereology cannot be performed on paraffin section cut at this thickness. This approach uses upper and lower guard zones so as to eliminate the counting of tissue elements that are only partially on the particular section under study. It is concerning that this approach is listed as applied to tissue sections of 5um.

5. How many tissue sections are analyzed per brain? What is the spacing between sections? This information is important for the reader to be able to evaluate the depth to which analysis was applied across the lesion. This number of tissue sections per animal is not given for any of the quantification approaches, except for the infarct size quantification. The text mentions 3-6 sections per animal. This is a large range and would generate substantial variation in the amount of data in each animal that is compared.

Minor Points

Line 348 reads-“ As microglia, also astrocytes in the ischemic core degenerated during the first couple of days”- this is hard to understand and there is no evidence for this.

The Methods section only details the elevated body swing test. The details of the other tests needs to be described, as these are core methodologies in the manuscript.
